# *Growing Slowly 1* locus encodes a PLS-type PPR protein required for RNA editing and plant development in Arabidopsis

**DOI:** 10.1093/jxb/erw331

**Published:** 2016-09-26

**Authors:** Tingting Xie, Dan Chen, Jian Wu, Xiaorong Huang, Yifan Wang, Keli Tang, Jiayang Li, Mengxiang Sun, Xiongbo Peng

**Affiliations:** ^1^State Key Laboratory for Hybrid Rice, College of Life Sciences, Wuhan University, Wuhan 430072, China; ^2^State Key Laboratory of Plant Genomics and National Center for Plant Gene Research (Beijing), Institute of Genetics and Developmental Biology, Chinese Academy of Sciences, Beijing 100101, China

**Keywords:** *ABI5*, mitochondria, pentatricopeptide repeat proteins, RNA editing, root.

## Abstract

Most pentatricopeptide repeat (PPR) proteins are involved in organelle post-transcriptional processes, including RNA editing. The PPR proteins include the PLS subfamily, containing characteristic triplets of P, L, and S motifs; however, their editing mechanisms and roles in developmental processes are not fully understood. In this study, we isolated the *Arabidopsis thaliana Growing slowly 1* (*AtGRS1*) gene and showed that it functions in RNA editing and plant development. Arabidopsis null mutants of *grs1* exhibit slow growth and sterility. Further analysis showed that cell division activity was reduced dramatically in the roots of *grs1* plants. We determined that GRS1 is a nuclear-encoded mitochondria-localized PPR protein, and is a member of the PLS subfamily. GRS1 is responsible for the RNA editing at four specific sites of four mitochondrial mRNAs: *nad1-265*, *nad4L-55*, *nad6-103*, and *rps4-377*. The first three of these mRNAs encode for the subunits of complex I of the electron transport chain in mitochondria. Thus, the activity of complex I is strongly reduced in *grs1*. Changes in *RPS4* editing in *grs1* plants affect mitochondrial ribosome biogenesis. Expression of the alternative respiratory pathway and the abscisic acid response gene *ABI5* were up-regulated in *grs1* mutant plant*s*. Genetic analysis revealed that *ABI5* is involved in the short root phenotype of *grs1*. Taken together, our results indicate that *AtGRS1* regulates plant development by controlling RNA editing in Arabidopsis.

## Highlight

The mitochondrial pentatricopeptide repeat protein AtGRS1 edits RNA at four sites and is critical for development. The abscisic acid response gene *ABI5* participates in the short root phenotype of *grs1*.

## Introduction

Pentatricopeptide repeat (PPR) proteins are a class of RNA binding proteins characterized by the presence of a degenerate 35-amino-acid repeat, the PPR motif, which is arranged in tandem 2–50 times (Small and Peeters, 2000). The PPR motif (P motif) has another two variants, namely the S (short) motif with a length of 31 amino acids and the L (long) motif with a length of 35–36 amino acids. Based on their motifs, PPR proteins are divided into two subfamilies: the P subfamily has only P motifs, and the PLS subfamily contains characteristic triplets of P, L, and S motifs. Most members of the PLS subfamily contain extra conserved domains at their C-terminus, and these are designated the E, E+, and DYW domains (Lurin *et al.*, 2004; Cheng *et al.*, 2016).

PPR proteins are involved in many aspects of RNA processing in mitochondria and chloroplasts, including RNA cleavage, splicing, editing, and translation, and play crucial roles in plant developmental processes and responses to environmental stresses (Andrés *et al.*, 2007; Zehrmann *et al.*, 2009; Liu *et al.*, 2010; Murayama *et al.*, 2012; Zhu *et al.*, 2012; Haili *et al.*, 2013; Mei *et al.*, 2014; Yang *et al.*, 2014; Hsieh *et al.*, 2015). RNA editing is an important step in the post-transcriptional control of organelle gene expression. Most RNA editing in plants results in the conversion of cytidine (C) to uridine (U) (Covello and Gray, 1989; Gualberto *et al.*, 1989; Hiesel *et al.*, 1989; Shikanai, 2006; Chateigner-Boutin and Small, 2010). In the mitochondria of Arabidopsis, approximately 500 C-to-U editing sites had been uncovered (Giegé and Brennicke, 1999; Bentolila *et al.*, 2005, 2008). The mechanism of the editing reaction puzzled researchers for many years, until the first PPR protein, CHLORORESPIRATORY REDUCTION 4, was found to be involved in chloroplast RNA editing (Kotera *et al.*, 2005). Since then, PPR proteins have been found to be involved in RNA editing and all the discovered trans-factors involved in RNA editing in plants belong to the PLS subfamily (Takenaka *et al.*, 2013; Shikanai, 2015). Although several PPR proteins target individual sites, some are found to recognize more than two and even as many as eight sites (Kim *et al.*, 2009; Zehrmann *et al.*, 2009, 2012; Zhu *et al.*, 2012; Glass *et al.*, 2015). Although recently bioinformatics, biochemical, and structural analyses have shown that PPR proteins recognize RNA in one-motif to one-nucleotide binding mode (Yagi *et al.*, 2013; Yin *et al.*, 2013; Barkan and Small, 2014), the mechanism of how a single PPR protein recognizes multiple target sequences still needs further investigation.

Mutations in many RNA-editing PPR proteins do not result in any evident developmental defect (Zehrmann *et al.*, 2009; Verbitskiy *et al.*, 2010; Härtel *et al.*, 2013), although some PPRs are important in development (Yu *et al.*, 2009; Koprivova *et al.*, 2010; Liu *et al.*, 2010; Murayama *et al.*, 2012; Haili *et al.*, 2013; Yang *et al.*, 2014). The relationship between mutant phenotype and RNA editing has not received much attention until recently. Mutations in PPR proteins involved in chloroplast RNA editing have been shown to impair chloroplast biogenesis (Yu *et al.*, 2009). Several reports have shown that an increase in reactive oxygen species (ROS) is responsible for the developmental defects observed in the mitochondrial RNA editing by those mutant PPRs (Liu *et al.*, 2010; Yang *et al.*, 2014). The nature of other signaling pathways linking PPRs involved in mitochondrial RNA editing and plant development remains largely unknown.

In this study, we analyzed the Arabidopsis T-DNA knockout mutant *grs1-1*, which displays a phenotype of slow growth and sterility. Genetic and molecular analysis indicates that the *GRS1* gene encodes a PPR protein. Further studies showed that GRS1 is required for the RNA editing of four mitochondrial transcripts. The upstream sequences of these editing sites share some conserved nucleotides. The lack of RNA editing at these sites leads to reduced levels of functional mitochondrial complex I and affects mitochondrial ribosome biogenesis. Abscisic acid (ABA) response gene *ABI5* but not ROS is involved in the short root phenotype in *grs1*.

## Materials and methods

### Mutant library construction and selection of *grs1-1*

We generated an Arabidopsis mutant library with T-DNA encoding *LAT52::EGFP*, a cell-autonomous pollen-specific reporter (Twell *et al.*, 1989; Sessions *et al.*, 2002), and a hygromycin-resistance gene. T-DNA mutagenesis was carried out on *qrt1* plants (Preuss *et al.*, 1994), where mature pollen grains maintain male meiotic products in tetrads (Supplementary Fig. S1A, B at *JXB* online). Hygromycin-resistant plants, heterozygous for a single locus T-DNA insertion, produced tetrads with two mutant pollen grains emitting green fluorescent protein (GFP) fluorescence, and two wild-type grains that did not display any GFP activity (Supplementary Fig. S1C, D). This simplified the process of determining whether a T2 plant was heterozygous (tetrads are two GFP+ to two GFP−, HYG resistant), homozygous (all four tetrad members are GFP+, HYG resistant) (Supplementary Fig. S1E, F) or wild-type (all four tetrads members are GFP−) for a T-DNA induced mutation.

For *grs1-1* selection, T1 seeds were obtained by self-pollination of hygromycin-resistant *grs1-1* plant and sown on 1/2 MS plates with hygromycin to select *grs1-1* seedlings. Thirty-two hygromycin-resistant seedlings were grown on soil and the pollen grains of each plant were visualized under a fluorescence microscope to determining whether a T2 plant was heterozygotes, homozygotes, or wild-type. T1 seeds were sown on 1/2 MS plates for germination.

### Plant materials and growth conditions

*Arabadopsis thaliana qrt1* (Preuss *et al.*, 1994) was used as a wild-type strain. The *grs1-1* allele was isolated from our mutant library with hygromycin resistance (Wu *et al.*, 2012, Supplementary data). The *grs1-2* (CS428796) and *gin1-3* lines were obtained from the Arabidopsis Biological Resource Center (ABRC; Ohio, USA). The mutant *abi5-1* (Liu *et al.*, 2012) was provided by Dr Lei Zhang (College of Life Sciences, Wuhan University). The transgenic line *pCyclinB1;1:Dbox-GUS* (Colon-Carmona *et al.*, 1999) was provided by Dr Jian Xu (Temasek Life Sciences Laboratory, Singapore). Seeds were surface-sterilized with 20% bleach for 10min, and washed three times with sterile distilled water. Seeds were stratiﬁed for 3 d at 4 °C and then sown on 1/2 MS plates with 1.0% (w/v) sucrose. To decrease the ROS level in seedlings, diphenyleneiodonium (DPI, 100 μM, Sigma) or reduced glutathione (GSH, 300 μM, Sigma) was added to the culture media. Agar plates were placed in a growth room with a photoperiod of 16h light/8h dark. For kanamycin selection, 50mg l^–1^ of kanamycin (Sigma) was supplemented to the media. Similarly, 50mg l^–1^ of hygromycin (Roche) and 10mg l^–1^L of sulfadiazin (Sigma) were added for hygromycin selection and sulfadiazin selection, respectively. Plants were grown on soil in a greenhouse under long-day conditions (16h light/8h dark) at 22 °C.

### Cloning of the T-DNA ﬂanking sequence and characterization of the *grs1-1* and *grs1-2* alleles

The T-DNA ﬂanking sequence in the *grs1-1* mutant was cloned by TAIL-PCR (Liu *et al.*, 1995). The authenticity of the cloned sequence was conﬁrmed by PCR using two pairs primers located around the T-DNA left border (*GRS1-T1*, TGGAACAAGTTCATCACGGTTTC; LB-S, CCAAAATCCAGTACTAAAATCCAG) and right border (*GRS1-T2*, ATTCATGGTTTGTGCATAAAAAGAG; RB-S, CGCGCGGTGTCATCTATG). For the *grs1-2* allele, the T-DNA site was conﬁrmed by PCR using the following primers: *GRS1*-RP, GTGAAAATGGGAGCAAAAGTG; and LB3, TAGCATCTGAATTTCATAACCAATCTCGATACAC.

### Vector construction and plant transformation

Plasmids P092, P093, and P094 were produced as described previously (Wu *et al.*, 2012; Yan *et al.*, 2016). To generate the p*GRS1*::*GRS1* complementation construct, a 3876-bp wild-type genomic sequence containing the *AT4G32430* gene, 1078-bp upstream of the ATG codon and 506-bp downstream of the TAG codon sequences, was PCR-amplified (primers: *GRS1-F1*, NNNNGGTACCTGATGTTTTGGGAGCGACTTC; and *GRS1-R1*, NNNNCTCGAGACCAAACTCATACCTTAAAGCCATC) from genomic DNA and was then cloned into the P092 plasmid with T-DNA encoding p*LAT52::DsRED* and a kanamycin-resistance gene (Supplementary Fig. S2C). To examine the subcellular location of GRS1, we amplified and cloned the *35S* promoters into P094 to generate the *35S::EGFP* construct. Then the *GRS1* ORF was amplified (primers: *GRS1-CDS1*, NNNNGGTACCATGACCCTTCTGAACTATCTACACTGT; and *GRS1-CDS2*, NNNNCTCGAGAACTGCAACTTTCCCC TCCAAATTCATC) from genomic DNA and cloned into the *35S::EGFP* plasmid to generate a *35S::GRS1-EGFP* construct. To produce the mitochondrial marker line, we amplified the *TagRFP-T* (Shibata *et al.*, 2010) and put it under the control of *35S* to generate *35S::RFP*. Then we amplified and cloned the 129-bp DNA fragment containing the mitochondria-targeted pre-sequence of the located F1-ATPase gene *At5g13450* (Robison *et al.*, 2009) (using primers *MITO-1*, NNNNGGTACCGCCACCATGGCTAATCGTTTCAGATCAGG; and *MITO-2*, NNNNCTGCAGTGTTTGAGCAGAAGCA GTTGCATAAG) into *35S::RFP* to generate the *35S::Mito-RFP* construct. To investigate the expression pattern of *GRS1*, the *GRS1* promoter was amplified (primers: *GRS1-F1* as above, and *GRS1-R2*: NNNNCTCGAGAGAAGCAAACTAGTCGGATTCTAATTC) and put upstream of GUS (β-glucuronidase) in P093 to generate p*GRS1*::*GUS*. All the gene constructs were transferred into *Agrobacterium tumefacien*s strain GV3101 and transformed into Arabidopsis plants by the ﬂoral dip method (Clough and Bent, 1998).

### Genotype analysis of the genomic complemented lines

To identify the genotype of the genomic complemented lines, the DNA of these plants was extracted and PCR analysis was conducted using three pairs of primers (S1+A1, S2+A1, S1+A2) (Supplementary Fig. S2B, C): Primer S1, CATCTGTAGGCAACAGTTTCATCAC located upstream of the T-DNA insertion site; Primer S2, CCAAAATCCAGTACTAAAATCCAG located around the T-DNA left border; Primer A1, CTCTTCTCTCGCTTTTTAAGTTGC located downstream of the *AT4G32430* gene and beyond the genomic fragment used for complementation; and Primer A2, TGACTTAGTTGATTTGGAGGGTG located downstream of the genomic fragment used for complementation.

### Histochemical analysis of GUS activity

For *pCYCB1;1:Dbox-GUS* staining, we crossed the *pCYCB1;1:Dbox-GUS* stable lines with *grs1-1* mutant plants. F2 seeds were obtained by self-pollination of F1 and sown on 1/2 MS plates with hygromycin to select seedlings with the *grs1-1* background. Individual F3 seeds were obtained by self-pollination of these seedlings and sown on 1/2 MS plates for germination. GUS activity analysis was performed with 8-d-old seedlings (with normal roots and short roots), and the lines with all normal roots with GUS activity were regarded as homozygous for *pCYCB1;1:Dbox-GUS*. The seedlings with short roots were regarded as homozygoous for both *pCYCB1;1:Dbox-GUS* and *grs1-1*.

The histochemical analysis of GUS activity was performed according to Vielle-Calzada *et al.* (2000). Plant tissues were incubated at 37 °C in GUS-staining solution [2mM 5-bromo-4-chloro-3-indolyl glucuronide (X-Gluc) in 50mM sodium phosphate buffer, pH 7.0] containing 0.1% Triton X-100, 2mM K_4_Fe(CN)_6_ and 2mM K_3_Fe(CN)_6_. The stained tissues were then transferred to 70% (v/v) ethanol solution. Samples were mounted with traditional clearing solution and placed under a microscope (Olympus) fitted with differential interference contrast optics for imaging.

### Analysis of subcellular localization of GRS1

The iPSORT Prediction program (Bannai *et al.*, 2002) predicted that GRS1 is targeted to the mitochondria. To conﬁrm its mitochondrial localization, transgenic plants containing the *35S::GRS1-EGFP* construct were crossed with a transgenic mitochondrial marker line expressing *35S::mito-RFP*. The petal cells of the F1 progeny were visualized using a FV1000 confocal laser-scanning microscope (CLSM; Olympus). GFP ﬂuorescence was detected with excitation at 488nm and emission at 510–530nm; red fluorescent protein (RFP) ﬂuorescence was detected with excitation at 568nm and emission at 590–620nm.

### Analysis of RNA editing

The status of Arabidopsis mitochondrial RNA editing in *grs1* plants was examined as described by Zehrmann *et al.* (2008). Total RNA was extracted from 20-d-old *grs1* and wild-type seedlings. Complementary DNA fragments of all mitochondrial transcripts containing RNA editing sites were ampliﬁed by RT-PCR. The primers used in this experiment are given in Supplementary Table S3. The ampliﬁed PCR products were directly sequenced and the results were compared to the corresponding DNA sequence for each transcript.

### Phenotypic characterization

For the determination of the root meristem size, root tips were excised from seedlings 8 d after germination, and examined with a differential interference contrast (DIC) microscope (Olympus).

### Measurement of ROS in roots

For nitrobluetetrazolium (NBT) staining to detect superoxides, seedlings were incubated in a reaction buffer containing 1mM NBT (Sigma-Aldrich) and 20mM K-phosphate at pH 6.0 for 20min. The seedlings stained by NBT were washed three times with water and then transferred to acetic acid:ethanol (1:3, v/v) solution. To enable 3, 3- diaminobenzidine (DAB) staining to detect H_2_O_2_, the seedlings were incubated in 0.3mg ml^–1^ DAB (Sigma-Aldrich) dissolved in 50mM Tris-HCl (pH 5.0) for 12h. The seedlings stained by DAB were washed three times with water, and were then examined in 10% glycerol with an Olympus microscope.

### Quantitative RT-PCR

Total RNAs of seeds before germination and 7-d-old seedlings were extracted using the RNAqueous^®^ Phenol-free total RNA Isolation kit (Ambion)according to the manufacturer’s protocol. After digestion with RNase-free DNase I (Promega), the first strand of cDNA was synthesized using oligo-dT and M-MLV reverse transcriptase (Invitrogen). Quantitative PCR analysis was performed using FastStart Essential DNA Green Master (Roche) on a CFX ConnectTM Real-Time System (BioRad). Each experiment was repeated three times and samples were normalized using UBQ10 expression. Data acquisition and analyses used Bio-Rad CFX Manager software; the relative expression levels were measured using the 2^(–∆∆Ct)^ analysis method and the error bars in the figures represent the variance of three replicates. The genes and the primers used for detection of the mRNA expression are listed in Supplementary Table S4.

### Detection of enzyme activity of complex I

Analysis of the NADP dehydrogenase activity of mitochondrion complex I was performed according to Wu *et al*. (015). Proteins of crude organelle extract from young seedlings were solubilized with 1% (v/v) digitonin and resolved by Blue Native-PAGE. After PAGE, the NADH dehydrogenase activity of complex I was visualized by incubation of the gel in the presence of 1mM nitroblue tetrazolium (NBT) and 0.2mM NADH in 0.05M MOPS (pH 7.6).

## Results

### *GRS1* plays an essential role in vegetative and reproductive development

**Fig. 1.**
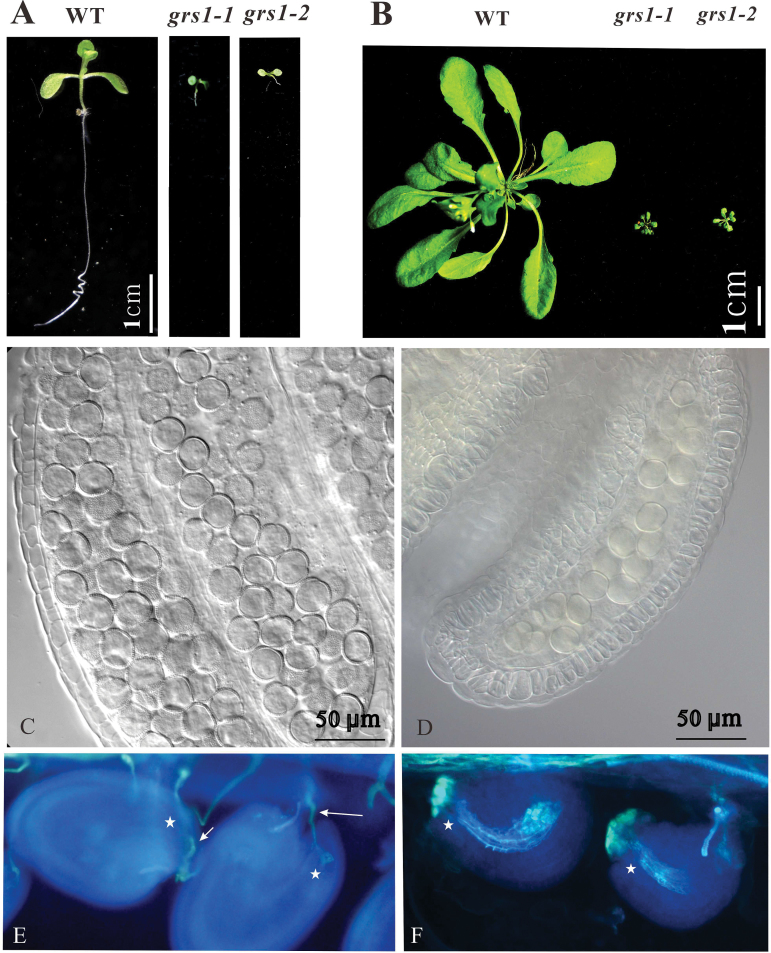
Several developmental processes are impaired in *grs1*. (A) Root growth of 8-d-old seedlings in wild-type (WT), *grs1-1*, and *grs1-2* plants. (B) Appearance of 35-d-old plants in wild-type, *grs1-1*, and *grs1-2*. (C, D) Anther of wild-type (C) and *grs1-1* (D) plants. The amount of pollen grains in *grs1-1* is much lower compared to the wild-type. (E, F) Aniline blue staining of pollen tube guidance in ovules. Ovules attract pollen tubes (indicated by arrows) in the wild-type (E), but no pollen tubes are observed in the ovules of *grs1-1* homozygous plants (F). The stars indicate the micropylar end of the ovules.

We generated an Arabidopsis mutant library to simplify the process of screening mutants whose homozygotes were lethal or exhibited growth retardation (Supplementary Fig. S1). One mutant displaying an extremely slow growth phenotype was isolated and named *growing slowly1* (*grs1-1*). When we analyzed the effect of *grs1-1* on plant development, we found that *grs1-1/+* heterozygous plants had no visible morphological abnormalities in vegetative and reproductive organs compared with wild-type plants. *grs1-1* homozygous plants, however, exhibited multiple phenotypes as shown in Fig. 1. Thirty-two hygromycin-resistant T2 plant were heterozygous *grs1-1*, suggesting that the *grs1-1* homozygotes are either lethal or exhibited growth retardation. T1 seeds of *grs1-1* were sown on 1/2 MS plates for germination and about 25% of 11-d-old seedlings showed an extremely slow growth phenotype (Supplementary Fig. S2A). The DNA of these slow-growth seedlings was extracted and PCR analysis confirmed that they were homozygous for *grs1-1* (Supplementary Fig. S2D)*. grs1-1* homozygous seedlings only survived on MS medium plates, and their vegetative growth was strongly affected (Fig. 1A, B). Opening the siliques of *grs1-1* two days after flowering revealed the absence of developed seeds. To determine which parent was responsible for the aborted phenotype, we performed reciprocal crosses of *grs1-1* and wild-type plants. Both females and males were found to be sterile in *grsl-1* mutant plants. Further analysis showed that the number of pollen grains in *grs1-1* was much lower than in the wild-type; female gametophyte development in *grs1-1* was also found to be retarded and did not appear to be able to attract wild-type pollen tubes into the ovules (Fig. 1C–F).

### Cell division is impaired in *grs1-1*

**Fig. 2.**
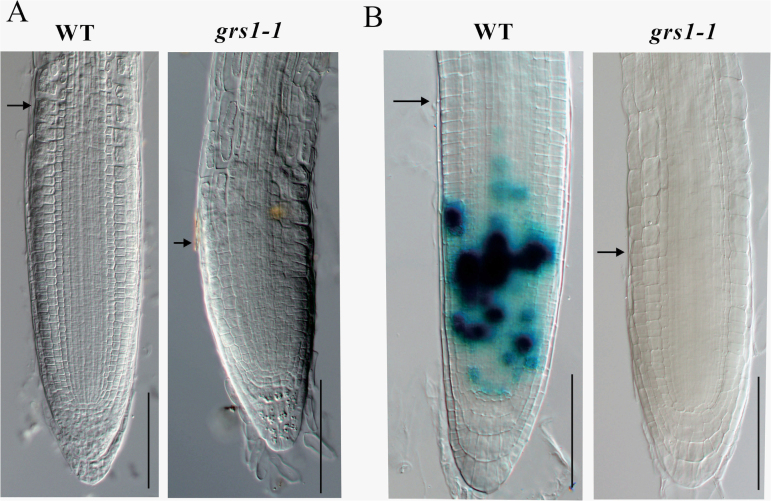
The activity of the root meristem division is reduced in *grs1-1*. (A) The root meristematic zone of 8-d-old wild-type (WT) plants is much longer than that of *grs1-1* plants. (B) Expression of *pCyclinB1;1:Dbox-GUS* in the meristematic zone of 8-d-old WT and *grs1-1* seedlings. Arrows indicate the boundary between the root meristematic and elongation zone. Scale bars = 100 µm.

After germination, the growth rate of the primary root was dramatically reduced in *grs1-1* plants compared to the wild-type. To determine the cellular basis for the observed defects in the root development of *grs1-1* plants, we examined the size of the root meristem in seedlings 8 d after germination. It was observed that the size of root meristem in *grs1-1* was much shorter than that of the wild-type (Fig. 2A). To further substantiate the role of *GRS1* in controlling root cell division, we crossed *pCyclin B1;1:Dbox-GUS* stable lines (Colon-Carmona *et al.*, 1999) with *grs1-1* mutant plants. The *pCyclin B1;1:Dbox-GUS* reporter allows the visualization of cells at the G2-M phase of the cell cycle, and thus to monitor mitotic activity in the root meristem (Colon-Carmona *et al.*, 1999). In contrast to the wild-type, we found that there was almost no GUS signal in *grs1-1* roots (Fig. 2B). The results indicate that the number of dividing cells was reduced dramatically in *grs1-1*compared to wild-type roots.

### Molecular characterization of *grs1-1*

**Fig. 3.**
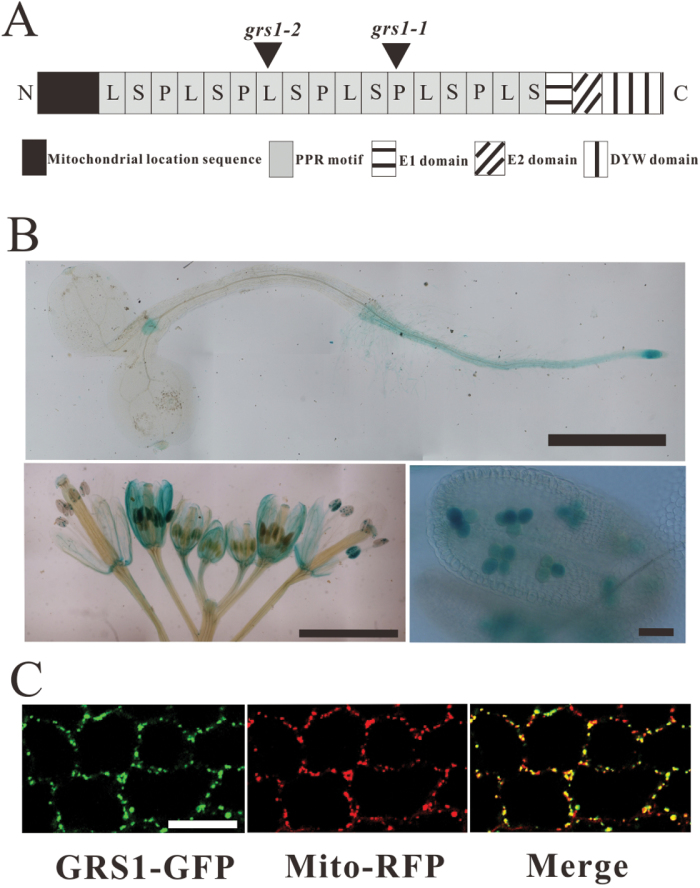
Structural features, expression patterns, and subcellular localization of GRS1. (A) Diagram showing the relative position of the T-DNA insertion in the *GRS1* gene and the structural features of the GRS1 protein. Various protein domains are indicated below the diagram. (B) GUS expression patterns in different plant parts of transgenic *proGRS1::GUS* lines. Top row: 7-d-old seedling. GUS signal is observed in root and shoot meristems. Scale bar = 5mm. Bottom row, left: an inflorescence, scale bar = 3mm. right: an anther with pollen grains, scale bar = 50 µm. (C) Localization of GRS1-GFP protein in the mitochondria. Petal cells of plant co-expressing GRS1-GFP and the mitochondrial marker Mito-RFP were examined with confocal laser scanning microscopy. From left to right: green fluorescent signal from GRS1-GFP; red fluorescent signal from the mitochondrial marker Mito-RFP; merged picture with green and red signals showing co-localization. Scale bar = 20µm.

Arabidopsis *grs1-1* plants were generated by T-DNA insertion with resistance to hygromycin. All the *grs1-1*/+ heterozygous plants were resistant to hygromycin, suggesting that the mutant phenotype was caused by a T-DNA insertion. We cloned the T-DNA ﬂanking sequence by using the thermal asymmetric interlaced polymerase chain reaction (TAIL-PCR) technique (Liu *et al.*, 1995). The *grs1-1* mutant was shown to carry a T-DNA insertion in the gene *AT4G32430* located 1325bp downstream of the ATG start codon (Fig. 3A, Supplementary Fig. S2B). Another allele containing a T-DNA insertion in the GRS1 gene, CS428796, was obtained from the Arabidopsis Biological Resource Center. We verified that the CS428796 mutant carries a T-DNA insertion in the *AT4G32430* gene at 850bp downstream of the ATG start codon (Fig. 3A). We then renamed the CS428796 allele *grs1-2.* Homozygous *grs1-2* plants were found to phenocopy *grs1-1* homozygous plants (Fig. 1A, B).

To conﬁrm that the *grs1-1* mutant phenotypes were indeed caused by knockout of the *AT4G32430* gene, we performed a complementation test with the genomic sequence of *AT4G32430*. Fifty-nine T1 transgenic plants were screened on double-resistance plates with hygromycin and kanamycin (for the transformed genomic sequence). Among them, eleven plants were homozygous for *grs1-1*. All these *grs1-1* homozygous plants carrying the fragments of the exogenous genomic sequence (resistance to kanamycin) showed no obvious differences compared to the wild-type, and were named the genomic complemented lines (homozygous for *grs1-1*, heterozygous for exogenous genomic fragment) (Supplementary Fig. S2A). Genotype analysis confirmed the genomic complemented lines contained both the mutated *grs1-1* version and expression of the wild-type version (Supplementary Fig. S2D). These results indicate that the *AT4G32430* gene can successfully complement the *grs1-1* phenotype. The *AT4G32430* gene was therefore renamed as *GRS1*.

### *GRS1* encodes a mitochondria-targeted pentatricopeptide repeat protein

To investigate the expression pattern of *GRS1*, we fused the *GRS1* promoter sequence to a GUS reporter gene, and transformed this construct into the wild-type. In seedlings, *GRS1*::GUS was preferentially expressed in the meristematic region of both roots and stems. In flowers, GUS activity was detected in the sepal, stigma, stamen, and pollen grains (Fig. 3B).

BLAST analysis identiﬁed *GRS1* as a member of the PPR family, more specifically belonging to the PLS subfamily. Thus, *GRS1* encodes a PLS-type pentatricopeptide repeat protein, as proposed by Lurin *et al.* (2004). It consists of six PPR-like S, six PPR-like L, and five P motifs with E1, E2, and DYW C-terminal extensions (Lurin *et al.*, 2004; Barkan and Small, 2014; Cheng *et al.*, 2016) (Fig. 3A, Supplementary Fig. S3, and Table S1). The iPSORT Prediction program (Bannai *et al.*, 2002) predicted that GRS1 is targeted to mitochondria and, indeed, GRS1-GFP was found to co-localize with the mitochondria-localized Mito-RFP (Fig. 3C), indicating that GRS1 is a nuclear-encoded mitochondrial protein.

### GRS1 is required for mitochondrial RNA editing

**Fig. 4.**
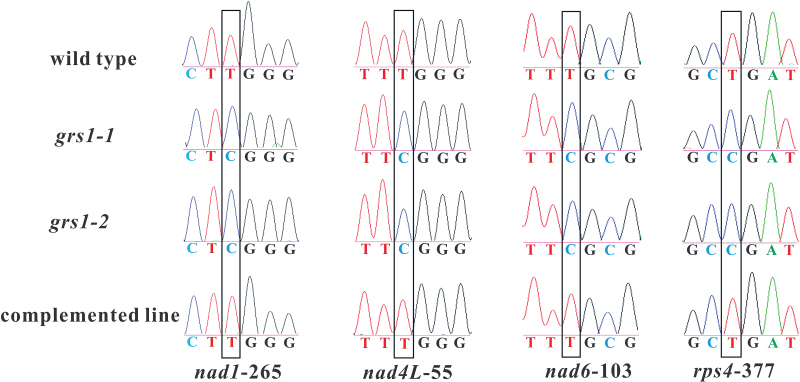
GRS1 is responsible for RNA editing of four sites in Arabidopsis mitochondria. Wild-type plants show that RNA editing of the mitochondrial editing sites *nad1*-265, *nad4L*-55, *nad6*-103, and *rps4*-377 is efficient, while in *grs1-1* and *grs1-2* these sites are not edited. Editing deﬁciencies were restored in the *grs1-1* complemented lines.

Since *GRS1* encodes a DYW-type PPR protein, we tested its involvement in mitochondrial RNA editing. We identiﬁed several unedited sites in the mitochondrial RNA in the *grs1-1* mutants. Our results revealed that C-to-U editing at the positions of *nad1*-265, *nad4L*-55, *nad6*-103, and *rps4*-377 was speciﬁcally blocked in the *grs1-1* plants. Editing of these four sites is also inhibited in *grs1-2* mutants (Fig. 4). The C-to-U editing in the *nad1* mRNA results in an arginine-to-tryptophane amino acid change (R89W) in the NAD1 protein. The C-to-U editing in the *nad4L* mRNA results in an arginine-to-tryptophane amino acid change (R19W) in the NAD4L protein. The C-to-U editing in the *nad6* mRNA results in an arginine-to-cystine amino acid change (R35C) in the NAD6 protein. The C-to-U editing in the *rps4* mRNA results in a proline-to-leucine amino acid change (P126L) in the RPS4 protein. Editing of the four mRNAs at these four editing sites was highly efficient in the wild-type, as shown by the detection of a single peak equivalent to the T nucleotide at these positions, whereas editing of these sites was totally abolished in *grs1-1* and *grs1-2* mutants (Fig. 4). Editing deﬁciencies of the mutant alleles were restored in the *grs1-1* complemented lines (Fig. 4). These results conﬁrmed that mutation in the *GRS1* gene was responsible for the defect of mitochondrial RNA editing in the *grs1-1* mutants.

### Complex I function and mitoribosomal biogenesis are impaired in *grs1-1* mutants

**Fig. 5.**
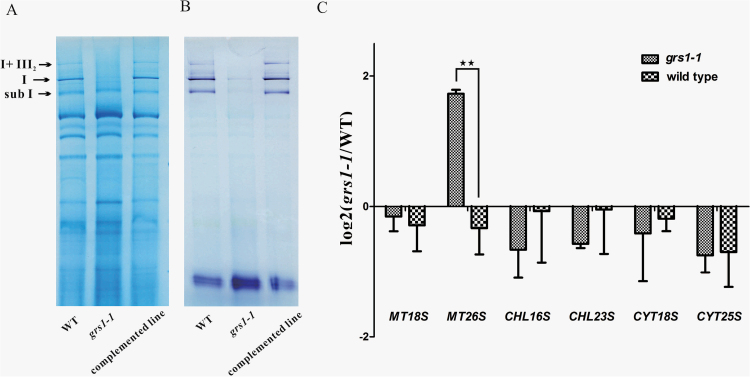
Complex I activity and mitoribosomal biogenesis are affected in *grs1-1* mutants. (A) Proteins of crude organelle extractions from young seedlings of wild-type (WT), *grs1-1*, and *grs1-1* complemented lines were stained by Coomassie blue. (B) In-gel assay of NADH dehydrogenase activity in WT, *grs1-1*, and *grs1-1* complemented lines. Activity of complex I could hardly be detected in *grs1-1*. The activity staining bands on the lower part of the gel correspond to the activity of the dehydrolipoamide dehydrogenase, which can serve as a loading control. I+ III_2_, mitochondrial complex I and complex III super-complex; I, mitochondrial complex I; sub I, mitochondrial sub-complex I. (C) Accumulation of rRNAs as a proxy for corresponding ribosomal subunits in *grs1-1* compared with wild-type plants. Levels of rRNA transcripts of large subunits and small subunits in mitochondrial, chloroplast, and cytosolic ribosomes are shown. The values obtained were averaged for three biological replicates, with error bars representing SD. Statistically significant differences between *grs1-1* and the wild-type are indicated: **P<0.01 (Student’s *t*-test).

The proteins NAD1, NAD4L, and NAD6 are components of the mitochondrial electron transport chain complex I (NADH dehydrogenase). Having observed that RNA editing of these genes was altered in *grs1-1* mutants and resulted in amino acid changes, we hypothesized that RNA editing defects of these transcripts may lead to complex I malfunction in *grs1-1* mutants. To test this hypothesis, we isolated crude mitochondria from seedlings of wild-type, *grs1-1* mutants, and *grs1-1* complemented lines. Separation of mitochondrial complexes by blue-native PAGE and NADH dehydrogenase activity staining showed that both protein levels and activity of complex I could barely be detected in *grs1-1* mutants (Fig. 5A, B).

Since the RPS4 protein is a component of the small subunit (SSU) of the mitoribosome, we tested whether the change in *RPS4* editing in the *grs1* mutants affects mitochondrial ribosome biogenesis. As rRNAs are unstable when unassembled, rRNA levels can serve as a marker for the accumulation of ribosomal subunits (Walter *et al.*, 2010; Kwasniak *et al.*, 2013). We determined the abundance of mitochondrial (mt 18S and mt 26S), chloroplast (chl 16S and chl 23S) and cytosolic (cyt 18S and cyt 25S) rRNAs. The mt 18S showed no evident difference between *grs1-1* and the wild-type, while a significant increase was observed for mt 26S rRNA in *grs1-1* plants compared to the wild-type (Fig. 5C), with the increased ratio of mt 26S to mt 18S indicating an imbalance between mitoribosomal subunits. The chl 16S, chl 23S, cyt 18S, and cyt 25S showed no obvious differences between *grs1-1* and the wild-type (Fig. 5C), suggesting the *grs1* mutation only affects mitochondrial ribosome biogenesis.

### An alternative respiratory pathway is activated in *grs1-1* mutants

**Fig. 6.**
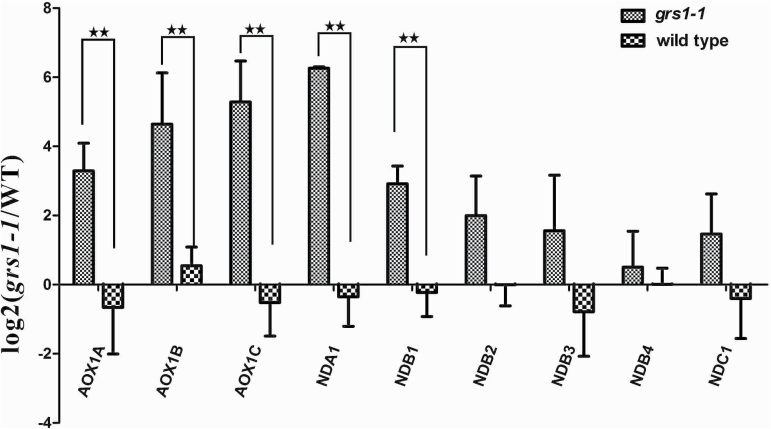
The alternative respiratory pathway is activated in *grs1-1*. The expression levels of alternative respiratory pathway genes in *grs1-1* increased signiﬁcantly relative to the wild-type. These genes include three alternative oxidases (AOXs) and six alternative NAD(P)H dehydrogenases (NDs). The values obtained were averaged for three independent experiments, with error bars representing SD. Statistically significant differences between *grs1-1* and the wild-type are indicated: **P<0.01 (Student’s *t*-test;).

Lack of complex I activities is known to result in elevated levels of an alternative respiratory pathway in Arabidopsis (Yuan and Liu, 2012). The components of this alternative respiratory pathway include several alternative NAD(P)H dehydrogenases (NDs) and alternative oxidases (AOXs). To determine whether *grs1-1* mutants had the same phenotype, we performed quantitative RT-PCR assays for the transcripts levels of six ND genes and three AOX genes in wild-type and *grs1-1* plants. As shown in Fig. 6, the expression levels of the nine examined genes in *grs1-1* increased signiﬁcantly relative to the wild-type. These results indicate that the alternative respiratory pathway is activated in *grs1-1*. *grs1-2* mutants had a similar phenotype with up-regulation of transcripts for alternative respiration compared with the wild-type. (Supplementary Fig. S4).

### The *grs1-1* mutant does not accumulate higher amounts of ROS than the wild-type

**Fig. 7.**
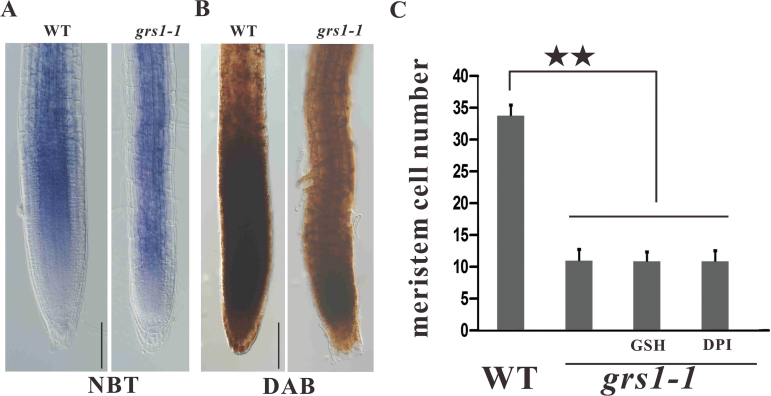
*grs1-1* mutants do not accumulate more ROS than the wild-type. (A) Nitroblue tetrazolium (NBT) staining for superoxide in primary root tips of wild-type and *grs1-1* plants. (B) 3, 3-diaminobenzidine (DAB) staining for H_2_O_2_ in primary root tips of wild-type and *grs1-1* plants. Scale bars = 100 µm. (C) Root meristem cell number in wild-type, *grs1-1*, and *grs1-1* with addition of reducing agents glutathione (GSH) or diphenyleneiodonium chloride (DPI). The values obtained were averaged for *n*>20, with error bars representing SD. Statistically significant differences are indicated: **P<0.01 (Student’s *t*-test;).

Reports have shown that impaired activity of the mitochondrial electron transport chain of complex I can cause a redox imbalance and increases in ROS accumulation, leading to the accumulation of more ROS in mutants than in the wild-type (Liu *et al.*, 2010; Yang *et al.*, 2014). We analyzed the ROS levels in *grs1-1* mutants and wild-type plants and showed that *grs1-1* mutants do not accumulate higher amounts of ROS than the wild-type (Fig. 7A, B). Consistent with these results, addition of the reducing agent glutathione (GSH) or diphenyleneiodonium chloride (DPI) was not able to complement the root growth defects of *grs1-1* mutant plants (Fig. 7C).

### *abi5* partially rescues the post-germination growth arrest of *grs1-1*

**Fig. 8.**
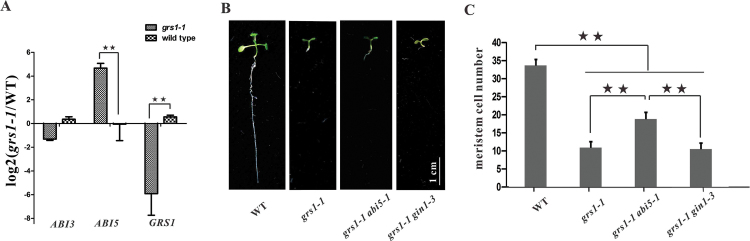
The *abi5-1* mutant partially rescues the post-germination growth arrest of *grs1-1* mutants. (A) Relative expression of *ABI3*, *ABI5*, and *GRS1* in wild-type and *grs1-1* plants. (B) Root growth of 8-d-old seedlings of wild-type, *grs1-1*, and *grs1-1 abi5-1* and *grs1-1 gin1-3* double-mutants. Scale bar = 1cm. (C) Root meristem cell number in wild-type, *grs1-1*, and *grs1-1 abi5-1* and *grs1-1 gin1-3* double-mutants. The values obtained were averaged for *n*>20, with error bars representing SD. Statistically significant differences are indicated: **P<0.01 (Student’s *t*-test;).

Since *grs1* mutant display defects in seed germination and post-germination growth, it is possible that the ABA signaling pathway is activated in these mutants. Given that the transcription factors ABI3 and ABI5 are key proteins in the ABA signaling pathway (Finkelstein and Lynch, 2000; Lopez-Molina *et al.*, 2001), expression of *ABI3* and *ABI5* was analyzed in *grs1-1* mutant and wild-type seedling plants 8 d after germination. Expression of *ABI5* was found to be significantly up-regulated in *grs1-1* mutants, whereas expression levels of *ABI3* were not significantly altered (Fig. 8A), implying that *ABI5*, but not *ABI3*, is activated in *grs1-1* mutants and is involved in the short-root phenotype. To test this hypothesis, the *grs1-1 abi5-1* double-mutant was generated, and it showed longer roots than those of the *grs1-1* mutants (Fig. 8B, Supplementary Table S2). While only about 10 cells could be observed in the meristems of in *grs1-1* mutants, approximately 20 cells were established in the meristem of *grs1-1 abi5-1* double-mutant plants (Fig. 8C). These results indicate that *abi5-1* partially rescues the post-germination growth arrest of the *grs1-1* mutants.

We then tested whether a decrease in the ABA content in *grs1-1* mutants can rescue the post-germination growth arrest of these plants. The *gin1-3* mutant line is a knockout allele of the ABA2 gene, one of the key genes involved in ABA synthesis However, the *grs1-1 gin1-3* double-mutant did not show any evident differences compared with the *grs1-1* single-mutant plants in post-germination growth (Fig. 8B, C).

## Discussion

### Putative cis-acting elements recognized by GRS1

Recently bioinformatics, biochemical, and structural analyses have shown that PPR proteins recognize RNA in one-motif to one-nucleotide binding mode (Kim *et al.*, 2009; Yagi *et al.*, 2013; Yin *et al.*, 2013; Barkan and Small, 2014). The major determinant is the amino acid at position 5 of the motif (Yagi *et al.*, 2013; Yin *et al.*, 2013; Barkan and Small, 2014; Cheng *et al.*, 2016). The second major determinant is at position 2 of the motif and position 35 of the following motif (Yagi *et al.*, 2013; Yin *et al.*, 2013; Barkan and Small, 2014; Cheng *et al.*, 2016). The site-specific RNA editing factors PPR and the RNA target sequences show optimal correlations when the PPR domains are aligned with the nucleotide sequences upstream of RNA editing sites up to the fourth nucleotide (nucleotide −4). The last S motif of GRS1 is accordingly positioned at the −4 nucleotides site of all the editing sites (Supplementary Fig. S3 and Table S1). In this way, the conserved A nucleotide at position −12 and G nucleotide at position −6 are consistent with the predictions of bioinformatics (Kim *et al.*, 2009; Yagi *et al.*, 2013; Yin *et al.*, 2013; Barkan and Small, 2014).

Cis-elements located between 20 to 25 nucleotides upstream and one to three nucleotides downstream of the edited C are known to be important in the context of RNA editing in mitochondria and plastids (Zehrmann *et al.*, 2009; Barkan and Small, 2014). When comparing the context of the four RNA sites edited by GRS1, five nucleotides are identical in addition to the edited C (Supplementary Fig. S3), suggesting that these positions are important for guiding editing through GRS1 in the mitochondria. These five nucleotides, however, are not sufficient to specify a unique site in the plant mitochondrial transcriptome. An *in silico* screen identified *NAD4*-403, another editing site with the same RNA context in the mitochondrial genome (Supplementary Fig. S3). *NAD4*-403 is edited normally in the wild-type and in the *grs1* mutant, confirming that the five shared nucleotide positions are not sufficient to guide editing through GRS1. More information may be provided by other nucleotides inside the context of RNA editing of the four sites to ensure GRS1 specifically binds to them. It was reported that PPR proteins distinguish purines from pyrimidines much better than they distinguish between C/U or A/G (Yagi *et al.*, 2014; Kindgren *et al.*, 2015). The conservation between these four sequences is better than shown when this is taken into account, with several other nucleotide positions, such as −4, −7, −9, −14, and −15, showing expected matches to the protein sequence in addition to the ones that have been indicated. The correlations of the amino acid codes in GRS1 and the diversity of its targeted RNA bases can offer more information for predicting whether a PPR protein can bind a particular RNA.

### Comparison of *grs1-1* plants with other Arabidopsis complex I mutant lines

Loss of GRS1 directly affects the editing of three components of complex I: nad1-265, nad4L-55, and nad6-103, which in turn impair the function of complex I. Most complex I mutants show a retarded growth phenotype, such as *ahg11* (Murayama *et al.*, 2012), *abo5* (Liu *et al.*, 2010), *abo8* (Yang *et al.*, 2014), *bir6* (Koprivova *et al.*, 2010), *css1* (Nakagawa and Sakurai, 2006), *indh* (Wydro *et al.*, 2013), *mtsf1* (Haili *et al.*, 2013), *nMat1* (Keren *et al.*, 2012), *nMat2* (Keren *et al.*, 2009), *nMat4* (Cohen *et al.*, 2014), *otp43* (de Longevialle *et al.*, 2007), *otp439* and *tang2* (Colas des Francs-Small *et al.*, 2014), *slg1* (Sung *et al.*, 2010), *slo2* (Zhu *et al.*, 2012), *slo3* (Hsieh *et al.*, 2015), and also as*smk1* (small kernel 1), which has been shown to be responsible for loss of editing of *NAD7*-448 transcripts in maize and rice (Li *et al.*, 2014).

The phenotype of *grs1-1* plants, however, cannot be fully explained by the loss of function of complex I. The defects observed in *grs1-1* plants are much stronger than those of mutants defective in complex I activity such as the *slo2* (Zhu *et al.*, 2012), *opt43* (de Longevialle *et al.*, 2007), *nMat1* (Keren *et al.*, 2012) and *ndufs4* mutants (Meyer *et al.*, 2009). Impaired activity of the mitochondrial electron transport chain of complex I can cause a redox imbalance and increases in ROS accumulation, leading to the accumulation of more ROS in mutants than in the wild-type (Liu *et al.*, 2010; Yang *et al.*, 2014); however, the *grs1-1* mutants do not accumulate more ROS than the wild-type. Consistent with these results, addition of GSH or DPI could not restore the root growth defects of *grs1-1* mutant plants. The results indicate that other signals must be responsible for the retarded growth phenotype observed in *grs1-1* plants.

ABA is a well-established key player in seed germination and post-germination growth. Furthermore, some reports have shown that mutations of PPR proteins result in mutant plants that are more sensitive to ABA than wild-type plants (Liu *et al.*, 2010; Murayama *et al.*, 2012; Yang *et al.*, 2014). Expression of *ABI5* was found to be significantly up-regulated in the *grs1-1* mutants compared to the wild-type plants, while expression of *ABI3* was not up-regulated in *grs1-1* mutant plants compared to the wild-type. The results indicate that the up-regulated expression of *ABI5* is independent of the ABA signal. The *grs1-1 abi5-1* double-mutant displayed higher root meristem cell numbers than the *grs1-1*single-mutant plants. The results indicate that *abi5-1* partially rescued the post-germination growth arrest of *grs1-1* mutant plants. The *grs1-1 gin1-3* double-mutant, however, could not partially rescue the post-germination growth arrest of *grs1-1* mutant plants. These findings suggest that *ABI5*, but not ABA, is involved in the post-germination growth arrest of *grs1-1* mutant plants. The mechanism through which *grs1-1* mutant plants activate *ABI5* remains an interesting question for future investigation.

Other factors must be involved in the root growth defects of the *grs1-1* mutant plants, since *abi5-1* only partially rescued their post-germination growth arrest. One possibility is that the mutation of GRS1 also impairs the function of mitoribosomes, leading to a dysfunction of mitochondria in addition to the loss of function of complex I. This scenario is found in *mcsf1* mutants, where the activity of complexes I and IV are both reduced, leading to severe defects in embryo development, which is arrested at the early globular stage (Zmudjak *et al.*, 2013).

## Supplementary data

Supplementary data are available at *JXB* online.

Figure S1. Rapid identification of heterozygous and homozygous mutants through pollen fluorescence.

Figure S2. Genomic complement fragment of *At4g32430* rescues the phenotype of *grs1-1*.

Figure S3. Putative coordination of PPR motifs of GRS1 and RNA nucleotides around the editing sites targeted by GRS1.

Figure S4. Relative expression of alternative pathway genes in wild-type and *grs1-2*.

Table S1. PLS repeat structure of *At4g32430*.

Table S2. The root length of 8-d-old seedlings.

Table S3. Primers used for RNA editing analysis.

Table S4. Primers used for quantitative RT-PCR.

Supplementary Data
