# Supplementary material for: Growing Slowly 1 locus encodes a PLS-type PPR protein required for RNA editing and plant development in Arabidopsis
Source: J Exp Bot. 2016 Sep 26;67(19):5687–98. doi: 10.1093/jxb/erw331 (PMC5066490; doi:10.1093/jxb/erw331)
Supplement: Supplementary Data [file supp_67_19_5687__index.html]

Growing Slowly 1 locus encodes a PLS-type PPR protein required for RNA editing and plant development in Arabidopsis — Growing Slowly 1 locus encodes a PLS-type PPR protein required for RNA editing and plant development in Arabidopsis — Supplementary Data 

# *Growing Slowly 1* locus encodes a PLS-type PPR protein required for RNA editing and plant development in Arabidopsis

## Supplementary Data

Data files

- Supplementary\_Figures\_S1\_S4\_Supplementary\_Tables\_S1\_S4.pdf - Supplementary Data
