# Supplementary material for: Growing Slowly 1 locus encodes a PLS-type PPR protein required for RNA editing and plant development in Arabidopsis
Source: J Exp Bot. 2016 Sep 26;67(19):5687–98. doi: 10.1093/jxb/erw331 (PMC5066490; doi:10.1093/jxb/erw331)
Supplement: Supplementary Data [file supp_erw331_Supplementary_Figures_S1_S4_Supplementary_Tables_S1_S4.pdf]

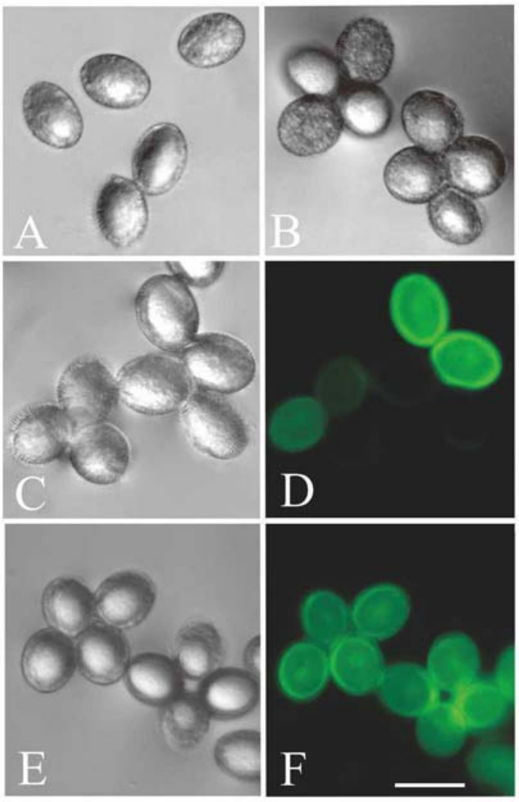

Figure S1 Rapid identification of heterozygous and homozygous mutant lines through pollen fluorescence. (A) *Arabidopsis thaliana* mature pollen grains of wild type. (B) The *qrt1* mature pollen grains maintain male meiotic products in tetrads. (C and D) Mature pollen grains of a heterozygous T-DNA insertion mutant. Two of four pollen grains from the same meiotic product are fluorescent; (E and F) Mature pollen grains of a homozygous T-DNA insertion mutant plant screened in T2 generation. The four pollen grains from the same meiotic product are fluorescent. Scale bar = 20µm.

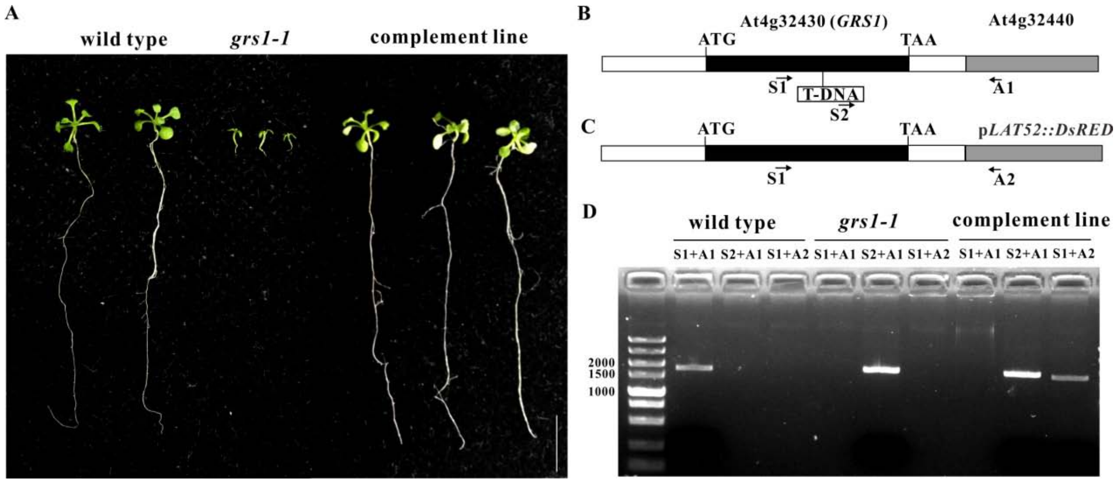

Figure S2 Genomic complementary fragment of At4g32430 rescues the phenotype of *grs1-1*.

A 11-day-old seedlings of wild type, *grs1-1* and the complemented line. Compared to wild type seedlings, *grs1-1* seedlings showed an extremely slow growth phenotype. Size of seedlings of the complemented line (homozygous for *grs1-1,* heterozygous for the genomic fragment) is similar to wild type seedlings. Scale bar = 1 cm.

B Diagram of the *GRS1* (At4g32430) genomic DNA and the insertion sites for the T-DNA in *grs1-1*. The location of primers S1, S2 and A1 used for genotyping are indicated by arrows.

C Diagram of the *GRS1* genomic complementary fragment and additional T-DNA element. The location of primers S1 and A2 used for genotyping are indicated by arrows.

D Genotypes of wild type, *grs1-1* and complemented line. Wild type shows a product of about 1700 bps (S1+A1). Homozygous *grs1-1* plants show a product of about 1800 bps (S2+A1). The complemented line shows two products of about 1800 bps (S2+A1) and 1700 bps (S1+A2).

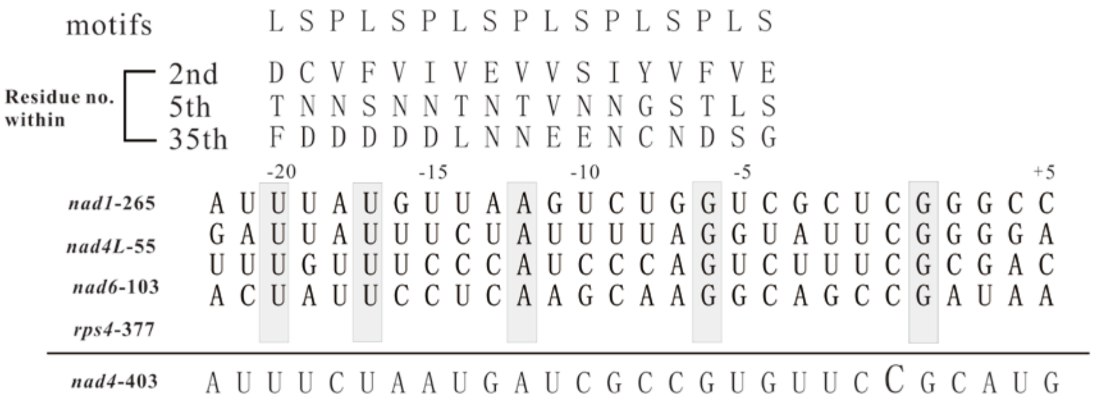

Figure S3 Putative coordination of PPR motifs of GRS1 and RNA nucleotides around the editing sites targeted by GRS1.

Top: PPR motifs of GRS1 and their corresponding residues at the 3nd, 6th of one PPR motif and 1th positions of the next motif (1’). Bottom: alignment of the putative cis-specificity regions around the RNA editing sites targeted by GRS1. Editing at *nad1-265, nad4L-55, nad6-103 and rps4-377* were inhibited in *grs1*. The edited C is shown in bold and the five nucleotides shared between the four sites are highlighted in grey. Another editing sites, *nad4-403*, share the same five nucleotides, but its editing is not affected in *grs1*. The last S motif of GRS1 is positioned at the -4 nucleotides of edited C.

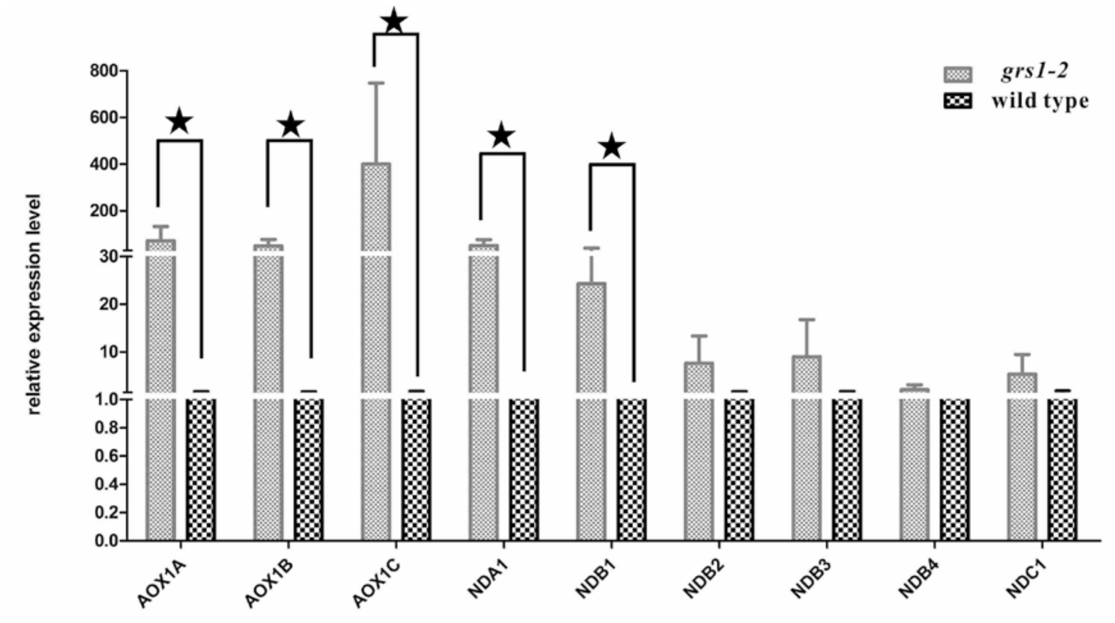

Figure S4 Relative expression of alternative pathway genes in wild type and *grs1-2*. The expression levels of alternative respiratory pathway genes in *grs1-2* increased significantly relative to WT. These genes include three alternative oxidases (AOXs) and six alternative NAD(P)H dehydrogenases (NDs). The values obtained were averaged for three independent experiments, with error bars representing SD. Statistically significant differences between *grs1-2* and wild type are indicated by asterisks (Student's t test; $P < 0.01$).

Table S1 PLS repeat structure of At4g32430

Location	Motif	Amino acid code			Class
		2nd	5th	35th	
76-110	MDEVTLCLALKACRGDLKRGCQIHGFST	D	T	F	L1
111-141	VCVSNAVMGMYRKAGRFDNALCIFENL	C	N	D	S1
142-173	VVSWNTILSGFDDNQIALNFVVRMKSAG	V	N	D	P1
174-208	AFTYSTALSFCVGSEGFLLGLQLQSTVVK	F	S	D	L1
209-239	LVVGNSFITMYSRSGSFRGARRVFDEMSF	V	N	D	S1
240-275	MISWNSLLSGLSQEGTFGFEAVVIFRDMM	I	N	D	P1
276-310	HVSFTSVITTCCHETDLKLARQIHGLCIKR	V	T	L	L1
311-341	LEVGNILMSRYSKCGVLEAVKSVFHQMS	E	N	N	S1
342-371	VVSWTTMISSNKDDAVSIFLNMRFDGVY	V	T	N	P1
372-406	EVTFVGLINAVKCNEQIKEGLKIHGLCIKT	V	V	E	L1
407-437	PSVGNSFITLYAKFEALEDAKKAFEDITFR	S	N	E	S1
438-471	IISWNAMISGFAQNGFSHEALKMFLSAAA	I	N	N	P2
472-508	EYTFGSVLNAIAFAEDISVKQGQRCHAHL	Y	G	C	L2
509-539	PVVSSALLDMYAKRGNIDESEKVFNEMS	V	S	N	S2
540-573	QFVWTSIISAYSSHGDFETVMNLFHKMIK	F	T	D	P2
575-610	LVTFLSVLTACNRKGMVDKGYEIFNMMI	V	L	S	L2
611-642	HEHYSCMVDMLGRAGRLKEAEELMSEV	E	S	G	S2
643-676	ESMLQSMLGSCRLHGNVKMGAKVAELA	S	Q	L	E1
677-710	SGSYVQMYNIYAEKEEWDKAAEIRKAM	G	V	K	E2
711-763	EAGFSWIDVGDTEGSLTMQGFSSGDKSHP KSDEIYRMVEIIGLEMNLEGKVAV	A	S	V	DYW

Table S2 The root length of 8-day-old seedlings

geneotype	Mean root length (mm)
wild type	42.5 ± 2.6
grs1-1	1.9 ± 0.4
grs1-1 abi5-1	3.7 ± 0.4
grs1-1 gin1-3	1.8 ± 0.3

Table S3 Primes for analysis of RNA editing

Gene	Primers (5' to 3')
atp1	atp1-F1:AGAGCTGCGGAACTAACGAATCTA
	atp1-R1: AAAGCATCTGGTTCCATTTTTCTT
atp6-1	atp6-1-F1: ATGCGACGAATCTTTTTGTTTGA
	atp6-R1: CCAAGCGAACCCACTTAAAATCTT
atp9	atp9-F1: ATGACAAAGCGTGAGTATAATTCTCAAC
	atp9-R1: TCAGAATACGAATAAGATCAAAAAGGCCATC
ccb203	ccb203-F1: TGGACACGGGGAGGGAGCAG
	ccb203-R1: CATAACATAACGGGGCGGGGTTG
ccb206	ccb206-F1: AACCAGCCTTGAAGTGAATG
	ccb206-R1: GAATCGAGGGTCCAGAAGAG
ccb256	ccb256-F1: CTACGCGCAAATTCTCATTGG
	ccb256-R1: GAGCGAGTGAACTAGGTTTTGGTA
ccb382	ccb382-F1: AATCTTGAAATGAGCACGAAATGTC
	ccb382-R1: CAGTACCTTTTAGTCGAGTGGCTTC
ccb452	ccb452-F1: TATGCAATTATGAACTCCACGGAAC
	ccb452-R1: ATAGAGCAGATGGTCCAACTACATAAC
cob	cob-F1: TGACTATAAGGAACCAACGATTCTC
	cob-R1: AGGTGTGATCAGTCTCATCCGTG
coxII	coxII-F1: GATTGTTCTAAAATGGTTATTCCTCAC
	coxII-R1: GGTTTGGGGGATTAATTGATTG
coxIII	coxIII-F1: TACCTCCCCACCAATAGATAGAGAC
	coxIII-R1: TTATGATTGAATCTCAGAGGCATTC
matR	matR-F1: GAAGTTTAGACCGCTCACAGTAG
	matR-R1: GTAGTTGAGTGTTCCTTCTTGTTC
nad1	nad1-F1: CCAGCTGAAATACTTGGAATAAT
	nad1-R1: AAAGGTGACTAAAAGACCAGAAAC
nad2	nad2-F1: CGTTCGGATCCTCCCACAC
	nad2-R1: TGAGTGCCATTTGATGAGTAACTG
nad3	nad3-F1: CCATTTTGTGCCCTATCACTTTAC
	nad3-R1: GCTGTAATGATGTCAGAATTTGCAC
nad4	nad4-F1: TTGCCATGTTGCACTAAGTTAC
	nad4-R1: CTTCAATGTTCGATTCTACTCTATG
nad4L	nad4L-F1: GTTCCCGAAATGGATCTTATCAAAT
	nad4L-R1: GGAGAGGAATGGTTAACCTTGAATG
nad5	nad5-F1: GATTATTATTCTTGACTTGACTTTG
	nad5-R1: CGATATGATGATTGGTTTAGGTAC
nad6	nad6-F1: GTAGATCGTGAGTGGGTCAGTCGTC
	nad6-R1: CCATGATACTTTCTGTTTTGTCGAG

nad7	nad7-F1: TCCCATGACGACTAGGAAAAGG
	nad7-R1: CCTATCTATCCACCTCTCCAAACAC
nad9	nad9-F1: GTTCCCAAGGACTAGCAAAATC
	nad9-R1: TCATTTTCAAATATAGTTGGGAGAC
*orf**	*orf**-F1: CATTAGCGGCTCATAAACCATG
	*orf**-R1: ATTTAGATCTCGGAGATCCCTTTC
orf25	orf25-F1: CCACCAAGCTCTCTCGAATG
	orf25-R1: TACGAATATGGATGGTAGAAAGATG
orf114	orf114-F1: CTAGCAGACTAATCACTAAATAGATAG
	orf114-R1: AGTCCTTGGAGCGTCTTTATG
orf240A	orf240A-F1: CCCTGAGTCGATCTCGCTG
	orf240A-R1: TGCGTAGTAGCGTTCTAAGATCAC
orfB	orfB-F1: ATCTAATCATGCCTCAACTGG
	orfB-R1: AGAATCATTTTATGCTTCCTTG
orfX	orfX-F1: ATCCTTCACTTTTAGCTTTGAATTAC
	orfX-R1: ATTGATAGTTACTTTGCCAGGTTC
rpl2	rpl2-F1: AGCACTTCCTCTATGGGCATTGTA
	rpl2-R1: TGCCTGCTTCTAATTGATGACTGG
rpl5	rpl5-F1: ATGTTTCCACTCAATTTTCATTAC
	rpl5-R1: GAGTTTCCCCCTCATCTTTT
rpl16	rpl16-F1: AAAATCGATTATGCTCCTG
	rpl16-R1: TACGACCACTGAACAAACTT
rps3	rps3-F1: AATCCGATTTCGGTAAGACTT
	rps3-R1: CGTTTCGGATATAGCACGTC
rps4	rps4-F1: GGCTGCTTAAAAAACTGATTC
	rps4-R1: TGTCCCTATGAGTGACTACGC
rps7	rps7-F1: GGGGGCTTGGATGGTGA
	rps7-R1: ATCTGAAATGCGCGAAACTT
rps12	rps12-F1: TCAATTGATTCGTCATGGTAGAGA
	rps12-R1: TCATATCGATTTGGGTTTTTCTGC
*rps14**	rps14-F1: ATGTCGTAGAAGCAAAATAGTAGA
	rps14-R1: TTATGCCCATCAAAGAACC
Ψ*sdh4**	Ψsdh4-F: ATGAAGGAACGAAAGAGTGG
	Ψsdh4-R: CCAAGATCTAATTTCGGGTC

Table S4 Primes for quantitative RT-PCR

Gene	Primers (5' to 3')
ABI5	ABI5-D1:ACTTGAGGATTTCTTGGTGAAGG
	ABI5-D2:CCTTGAAACACACCATAAAGCTG
ABI3	GTAAGACAACCGAGCGGACAA
	CATTCGCCGACGGAGAGTTAT
GRS1	GRS1-D1:AGATCAGGAAAGCAATGAGGAAG
	GRS1-D2:GAAACCTTGCATCGTTAAAGACC
MT18S	mt 18S rRNA-1: TCAGCATGCCGCGGTGAATATG
	mt 18S rRNA-2: TGCTTCGGGCGAAACCAATTCC
MT26S	mt 26S rRNA-1: TGCGAGGTGGGTAGTTTATC
	mt 26S rRNA-2: GGCAGGCTTATACCATTACG
CHL 16S	chl 16S rRNA-1: GCGTAGAGATCGGAAAGAAC
	chl 16S rRNA-2: GGGTATCTAATCCCATTCGC
CHL 23S	chl 23S rRNA-1: CTATAACGGTCCTAAGGTAGCG
	chl 23S rRNA-2: CTATTTCACCGAGCCTCTCTC
AOX1A	AOX1A-D1:GACGGTCCGTACGGTTTCG
	AOX1A-D2:CTTCTGATTCGCGTCCTCCT
AOX1B	AOX1B-D1:TGGAGACTTGAAGCTGATGC
	AOX1B-D2:TCTTTTAGCTCACGACCTTGG
AOX1C	AOX1C-D1:GCATCAAAGCAAGCGACATCC
	AOX1C-D2:TGGCTTCACGCCCCAATAAC
NDA1	NDA1-D1:GTCCGTGAGAGCAAGGAAGG
	NDA1-D2:GGCGAAGTGGAGGGGATATG
NDB1	NDB1-D1:CTGTGACTGATAAAGATATC
	NDB1-D2:GCCAACTGCATATACATTC
NDB2	NDB2-D1:CTGACTCTCTCAAAGAGTTCC
	NDB2-D2:CGATTTGAACTCTTCGATC
NDB3	NDB3-D1:GCAAATCCATCATATAGCAAC
	NDB3-D2:CAAGGGAGTGAAAGCAAAG
NDB4	NDB4-D1:TCAAGTCTTAAGGGCACAACA
	NDB4-D2:CGGAAGAGAGAGGCTCTCTCG
NDC1	NDC1-D1:TCCATAAAGAGAGCAAGCAAC
	NDC1-D2:CGAATCACCTAATGCAAATATC
UBQ10	UBQ10-D1: GGCCTTGTATAATCCCTGATGAATAAG
	UBQ10-D2: AAAGAGATAACAGGAACGGAAACATAGT
